# Fluorescence lifetime imaging ophthalmoscopy adds the retina to cortical pathology for visual dysfunction in neurodegenerative diseases

**DOI:** 10.3389/fneur.2025.1659264

**Published:** 2025-10-23

**Authors:** Jirat Nimworaphan, Daniel M. Markowitz, Robert C. Sergott

**Affiliations:** ^1^Department of Ophthalmology, Faculty of Medicine Vajira Hospital, Navamindradhiraj University, Bangkok, Thailand; ^2^William H. Annesley Jr. EyeBrain Center, Vicky and Jack Farber Neuroscience Institute, Thomas Jefferson University, Partnered with Wills Eye Hospital, Philadelphia, PA, United States; ^3^Drexel University College of Medicine, Philadelphia, PA, United States

**Keywords:** fluorescence lifetime imaging ophthalmoscopy, neurodegenerative diseases, Alzheimer’s disease, Parkinson’s disease, multiple sclerosis

## Abstract

Because neurodegenerative diseases such as Parkinson’s and Alzheimer’s Diseases [AD and PD] as well as the progressive forms of multiple sclerosis [MS] are invariably associated with clinically significant cortical symptoms such as language difficulties, motor skill deficits and cognitive impairments, especially memory, a tacit assumption evolved that visual disorders related to cortical dysfunction must localize only to the temporal, parietal and occipital lobes. Based upon our current understanding, retinal changes in MS are most likely secondary to optic neuropathy, whereas in AD and PD, they appear to represent primary retinal changes. The paradigm was reinforced by the lack of retinal findings using ophthalmoscopy. Spectral domain optical coherence tomography [OCT], optical coherence angiography [OCT-A], and fundus autofluorescence [FAF] have challenged this creed by uncovering structural changes within the retina over and above what can occur as a consequence of optic neuropathy in the case of MS. Still, definitive diagnostic and prognostic data have yet to emerge. Fluorescence lifetime imaging ophthalmoscopy [FLIO], a non-invasive, non-contact, painless imaging technology, measures nanosecond lifetimes of endogenous retinal fluorophores, some of which are linked to mitochondrial activity. Therefore, FLIO is a metabolic, not a structural imaging modality. Because mitochondrial dysfunction occurs in many neurodegenerative diseases, FLIO offers a unique strategy for investigating retinal metabolism in AD, PD, and MS. This article reviews the basic biomedical engineering of FLIO and reports preliminary data from these diseases, correlated with disease duration. These functional *in vivo* data are consistent with retinal metabolic changes in AD, PD, and progressive MS that were *“hiding in plain sight”* from structural examinations.

## Introduction

Certainly, in its earliest clinical stages and sometimes with more advanced neurodegenerative diseases, cortical visual dysfunction remains an elusive diagnosis. Despite precise localization through advanced magnetic resonance imaging [MRI] technologies (1–4), patients’ presenting symptoms are quite imprecise, simulating common ophthalmic disorders including refractive errors, keratitis sicca, cataracts, glaucoma, and maculopathies including macular degeneration and diabetic retinopathy.

In comparison to acute cerebral infarctions, both common and rare neurodegenerative conditions are associated with non-specific, non-diagnostic MRI findings of cortical atrophy often reported as “age-related” (1–5). Moreover, recent questions have emerged asking if the cortical dysfunction correlates with all the patients’ visual symptoms, especially decreased low contrast visual acuity.

Localizing and diagnosing the cause of visual symptoms can be challenging when the optic nerves and retinas appear normal on direct and indirect ophthalmoscopy, as well as on intravenous fluorescein angiography. However, a normal fundus does not exclude significant retinal and optic nerve dysfunction.

Spectral domain optical coherence tomography [SD-OCT] has generated many reports of abnormalities in both Alzheimer’s and Parkinson’s Diseases (6–19), but no definitive, easily identified, retinal structural imaging biomarkers have emerged to establish an authoritative diagnosis prior to clinically obvious dementia or impaired motor function. Promises for an earlier, more straightforward diagnosis are emerging with both blood and cerebrospinal fluid assays for abnormal proteins (20–26), but costs are a deterrent to widespread evaluation of large numbers of patients. Positron emission tomography [PET] scanning does offer definitive results (23, 27–30), however, accessibility is often restricted due to cost, insurance coverage limitations, and availability, so most patients continue to receive diagnoses primarily based on cognitive testing.

The era of metabolic imaging of the retina using fluorescence lifetime imaging ophthalmoscopy [FLIO] is developing rapidly. Although not yet widely available, FLIO performs non-invasive, painless, rapid imaging to produce both qualitative and quantitative data about the metabolic health of the retina (31–40). FLIO offers insights into visual symptoms and holds promise as potential biomarkers for earlier diagnosis and disease monitoring, even before clinical symptoms appear (34, 35, 37, 38). Since the brain and retina are linked *in utero* by a common neuro-ectodermal origin, specifically the diencephalon, a valid rationale emerges that pathological processes in the brain may also involve the retina (10, 41–44).

Because more and more data are emerging about FLIO and neurodegenerative diseases (35, 45–48), this article will review the technology behind FLIO and report longitudinal data with FLIO correlated with disease duration in relapsing–remitting and secondary progressive multiple sclerosis, Parkinson’s, and Alzheimer’s Diseases.

### Patient report of the effect of Alzheimer’s disease on visual function: cortical or retinal or both?

**Figure 1:**
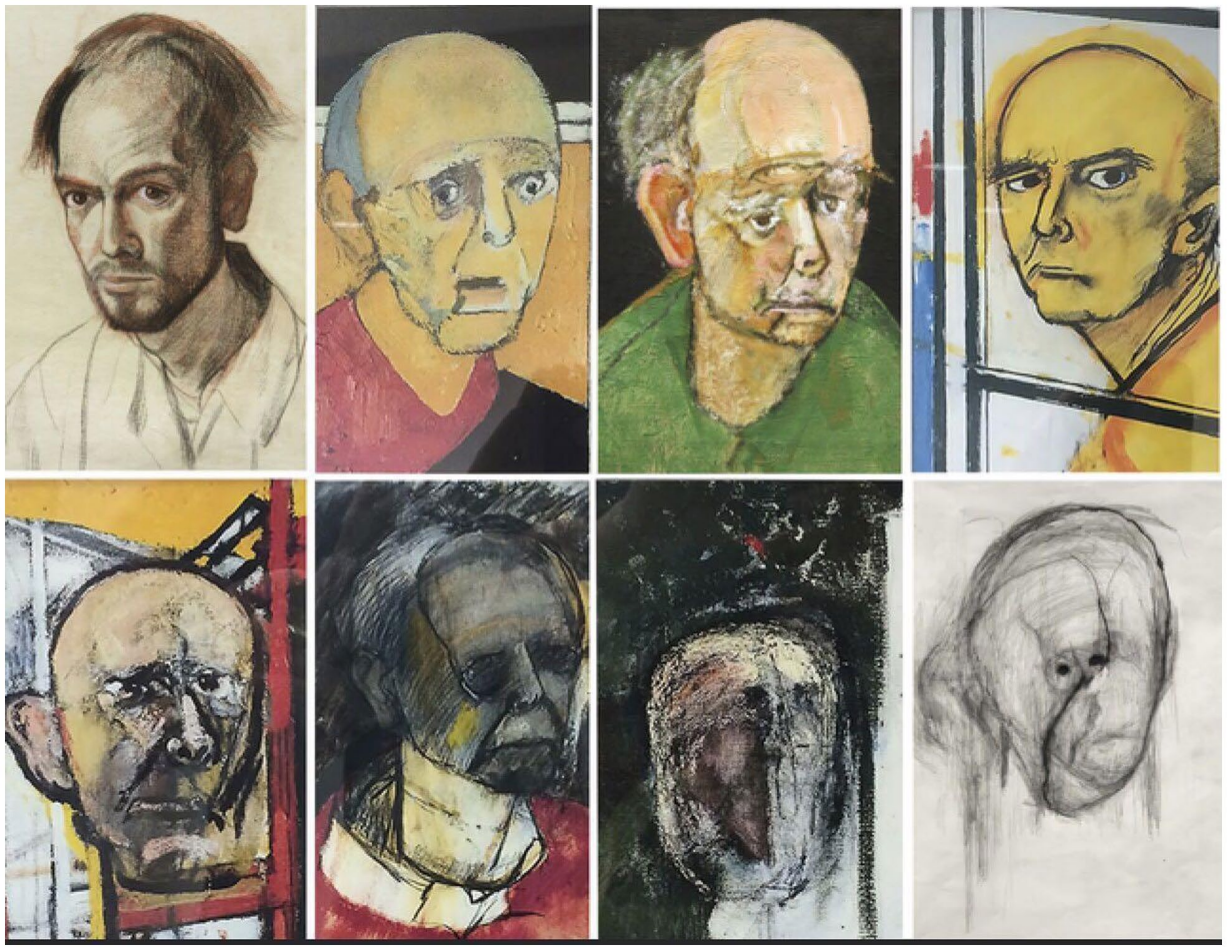
After being diagnosed with Alzheimer’s Disease, artist William Utermohlen, at the urging of his nurse, created a self-portrait each year until he was no longer able to draw. The first portrait on the upper left was drawn in 1995, and the last on the lower right was in 2001. He died in 2007. Adapted from Crutch et al. (49).

Born in South Philadelphia in 1933, William Utermohlen, an artist, created self-portraits each year after his diagnosis of Alzheimer’s Disease until he was no longer able to draw (49). His serial drawings in Figure 1 reveal a constellation of visual disturbances, some of which are certainly cortical in origin, with distortion of perspective and a lack of stereopsis. However, some details of his face disappear over the years in a pattern that may be consistent with central and paracentral scotomas but not resembling a homonymous hemianopia. These scotomas possibly localize to the maculae, plus his portraits reveal loss of some color perception, a finding that may localize to macular cone dysfunction as well as cortical involvement with the perception of color. Mr. Utermohlen died in London in 2007, no longer being able to draw his self-portrait for the last 6 years of his life (50).

In standard diagrams of the regions of the brain affected by Alzheimer’s, the retinas are never included, likely because of the lack of ophthalmoscopic findings in the retina and optic nerve. However, not seeing a process in the ophthalmoscope does not exclude pathological changes below the level of resolution of the ophthalmoscope.

**Figure 2:**
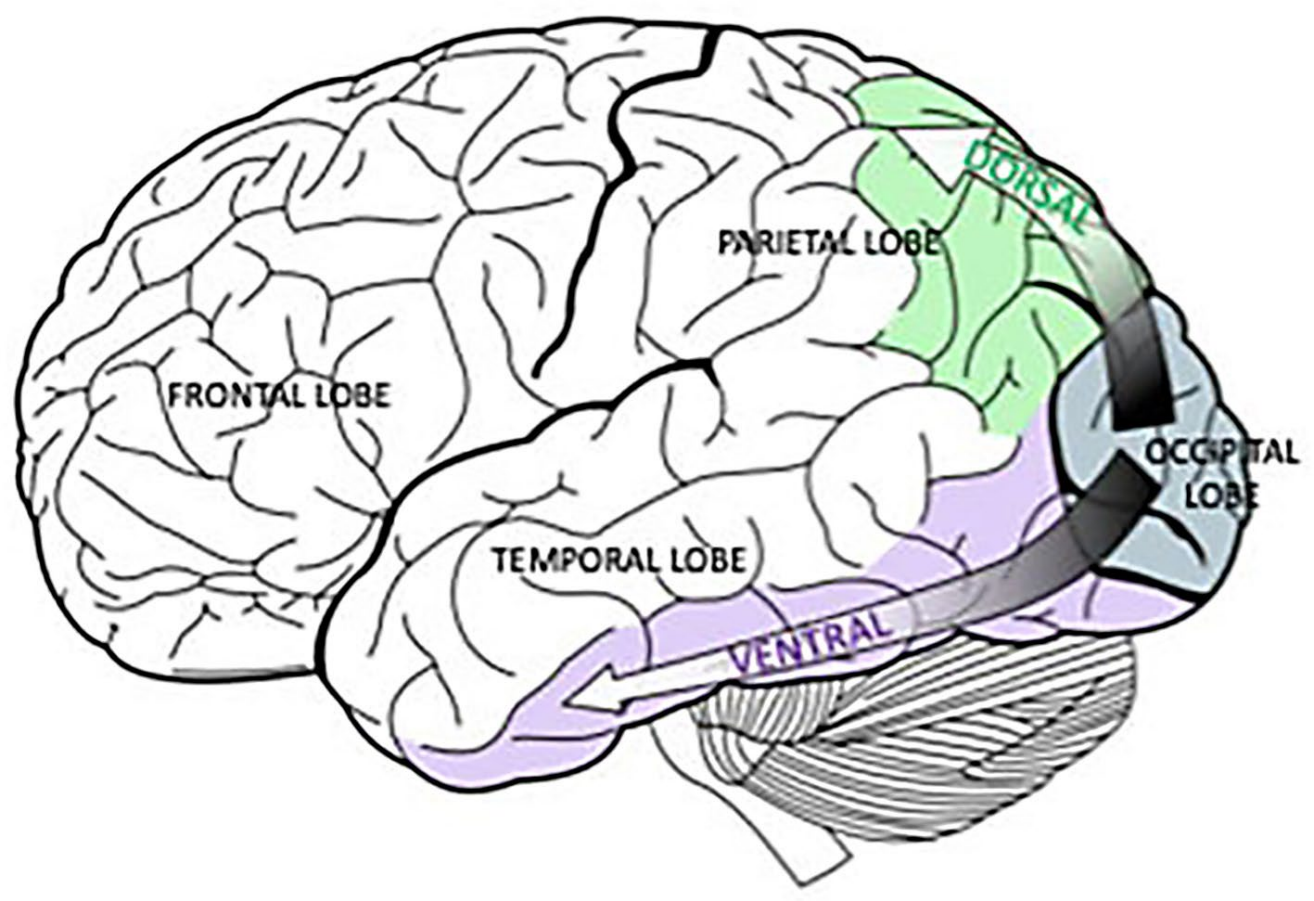
Originating in the occipital lobes, the green, dorsal pathway goes to the parietal lobe and is associated with spatial processing. In a similar way, the purple, ventral pathway connects the occipital lobes and the temporal lobe and is linked to object recognition*. Selket/Wikimedia Commons, GNU Free Documentation License, CC BY-SA 3.0.* https://solportal.ibe-unesco.org/articles/building-a-brain-that-can-read-part-1-sound-and-sight/.

Figure 2 provides precise neurological localization for pathological changes for the “what is it” symptom [ventral pathways] and the “where/how” symptoms [dorsal pathways], both originating from the occipital lobes. But do these areas of the afferent visual system explain all of the abnormalities in Mr. Utermohlen’s portraits and other patients suffering from Alzheimer’s Disease?

### Retinal structural findings in Alzheimer’s disease

In recent years, clinicians and clinical researchers are reporting more and more retinal structural abnormalities in patients with Alzheimer’s Disease (AD).

The retinal findings in patients with Alzheimer’s Disease follows:

1. Amyloid Beta (Aβ) Deposition (7, 12, 13, 17, 18, 35, 51–57):

- Aβ plaques, a hallmark of AD in the brain, are also found in the retina of AD patients.
- These deposits are often observed in the ganglion cell layer (GCL), inner nuclear layer (INL), and inner plexiform layer (IPL).
- The presence of retinal Aβ deposits can correlate with brain Aβ accumulation and disease severity.

2. Retinal Nerve Fiber Layer (RNFL) Thinning (6–10, 12, 55–57):

- The RNFL, composed of axons of retinal ganglion cells, shows thinning in AD patients, particularly in the superior and inferior quadrants.
- This thinning suggests loss of retinal ganglion cells and their axons, which may contribute to abnormal visual information to the temporal, parietal, and occipital lobes.

3. Ganglion Cell Layer (GCL) Thinning (10–13, 55–57):

- The GCL is also observed to be thinner in AD patients.
- This thinning reflects the loss of retinal ganglion cells, which are crucial for transmitting visual information to the brain.

4. Microglial Activation (53, 58–60):

- Microglia, the immune cells of the central nervous system, are activated in the retinas of AD patients.
- Activated microglia can contribute to neuroinflammation and potentially exacerbate neuronal damage.

5. Vascular Changes (13–19, 61):

- Alterations in the retinal vasculature, including reduced vessel density and increased foveal avascular zone (FAZ), have been reported in AD patients.
- These vascular changes may reflect similar changes occurring in the brain’s microvasculature.
- Dr. Fekrat and Dilraj S. Grewal, MD, also at Duke University, recently reported on OCT and OCTA findings in the superficial capillary plexus in eyes of patients with mild cognitive impairment (MCI) and AD (16). They found reduced macular vessel density, perfusion density, and ganglion cell-inner plexiform layer thickness in eyes with AD compared to those with MCI and normal controls.

6. Melanopsin-containing Retinal Ganglion Cell (mRGC) Loss (62–64):

- A specific subpopulation of retinal ganglion cells, the mRGCs, appear to be vulnerable in AD.
- These cells play a role in regulating circadian rhythms, and their loss may contribute to sleep disturbances and circadian dysfunction observed in AD.

7. Tau Pathology (8, 54, 65–67):

- Hyperphosphorylated tau, another hallmark of AD, has also been found in the retina of AD patients.
- The role and distribution of tau pathology in the retina, as well as its relationship to Amyloid-*β* (Aβ) accumulation, have been identified in the retina for neurodegeneration-associated disorders like Alzheimer’s disease (AD) and glaucoma.

### FLIO–metabolic imaging of the retina and the principles of fluorescence lifetimes

Over the past decade, university researchers have collaborated with industry to prototype and refine methods for assessing the metabolic environment of the retina, one of which is fluorescence lifetime imaging ophthalmoscopy [FLIO]. FLIO is the *in vivo*, clinical application of the well-validated research technology called fluorescence lifetime imaging microscopy [FLIM]. While most of the currently published FLIO data have been generated to date using the Heidelberg Engineering Spectralis-based device to measure fluorescence lifetimes, other groups have developed different *in vivo* retinal imaging systems to measure metabolic activity (36, 40, 68–73). The growing interest in retinal metabolic imaging will add a functional dimension of imaging and understanding of disease to established structural imaging modalities in a clinical setting.

The *in vivo* metabolic imaging method of FLIO complements high-resolution optical coherence tomography in a similar way that positron emission tomography [PET] scanning uses radioactive tracers to evaluate tissue function, thereby adding metabolic data to magnetic resonance imaging [MRI] scanning. However, compared to PET scanning, FLIO leverages the endogenous fluorophores of the retina to make the previously invisible metabolic functions visible. Therefore, FLIO fulfills many criteria for an ideal imaging biomarker because it is non-invasive, non-contact, painless, and performed quickly, producing data that is immediately available for analysis, and without requiring radioactive materials or imaging contrast agents. Dilation of the pupils is the only “invasive” part of FLIO.

### Fluorescence lifetimes

G. G. Stokes, the Lucasian Professor of Mathematics at Cambridge University, where he spent his entire career, including graduation from Pembroke College, discovered the property of fluorescence, the ability of certain materials to convert invisible ultraviolet light into light of longer wavelengths within the visible spectrum (74–76). FLIM and FLIO apply Stokes’ magnificent discovery made in 1852 to the real world of medicine in the 21st century.

From Stokes’ elegant work, the concepts of fluorescence intensity and fluorescence lifetimes were born. Fluorescence intensity refers to the brightness of fluorescence or the amount of light emitted by a molecule or compound when it is excited by another light. Greater intensity usually means a greater concentration of a molecule or a fluorescently labeled target. Fluorescence intensity is one of the principles of flow cytometry. However, intensity measurements are subject to a variety of artifacts, including uneven illumination and photobleaching, as well as motion and optical aberrations (74–76).

**Figure 3:**
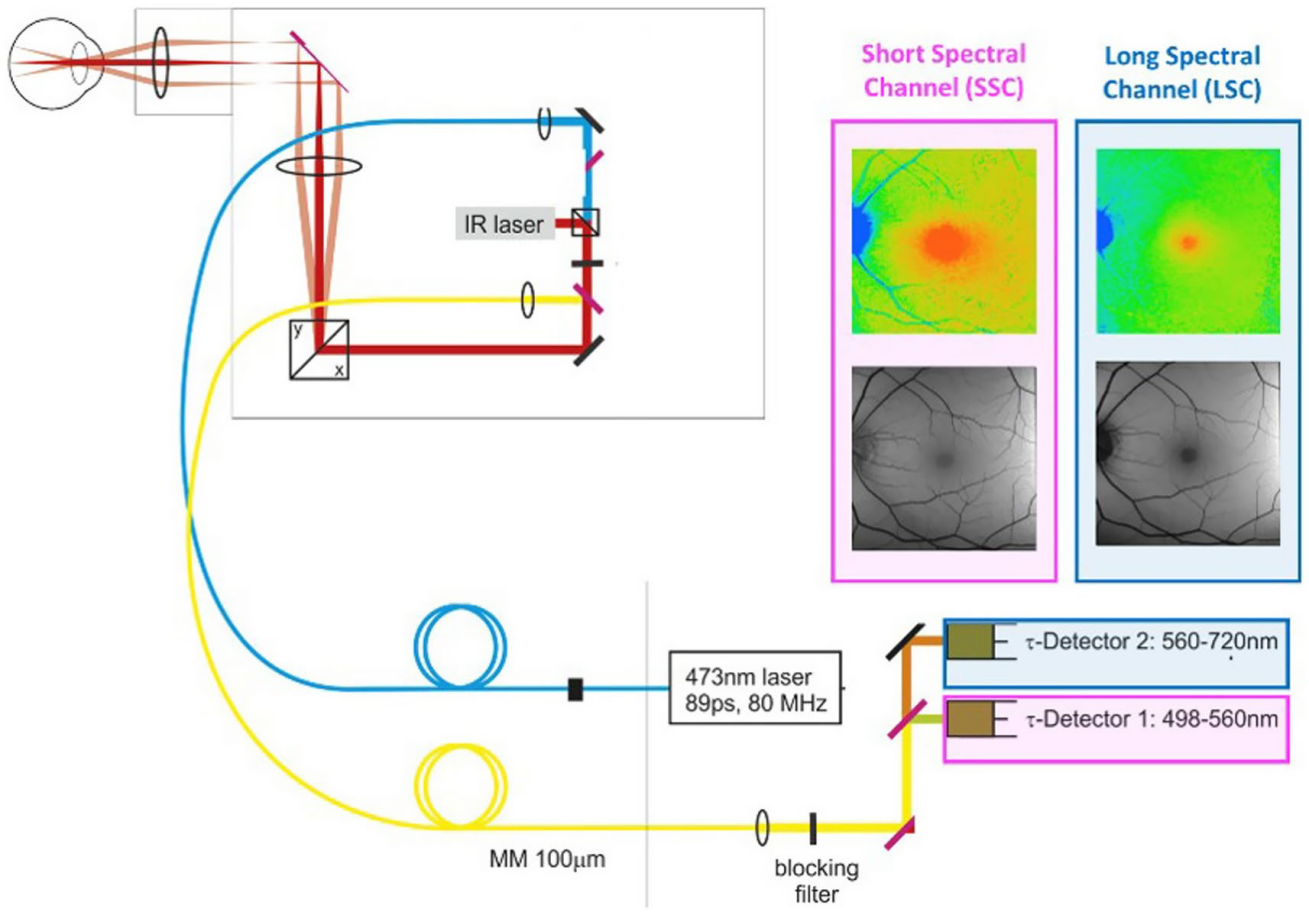
Retinal fluorophores are excited by a 473-nm pulsed laser. Emitted fluorescence is simultaneously detected in two distinct wavelength channels: a short spectral channel (SSC) between 498 and 560 nm and a long spectral channel (LSC) between 560 and 720 nm. The lifetime images are color-coded. An infrared eye-tracking system corrects eye movements.

In contrast, fluorescence lifetime imaging avoids many of the previously mentioned artifacts and records the average time a molecule spends in the excited state before returning to the ground state. These lifetimes are in the nanosecond range (10^−9^ s). Every molecule has a unique fluorescence lifetime signature so that individual molecules can be detected at different time points in a physiological or pathophysiological process. The current FLIO setup, which is only for investigational, not medical, care, purposes, is shown in Figure 3. In this system, the retinal fluorophores are excited by a 473-nm pulsed laser and the emitted fluorescence is simultaneously detected in two distinct wavelength channels: a short spectral channel (498–560 nm) and a long spectral channel (560–720 nm). Table 1 from Professor Christine Curcio’s research laboratory at the University of Alabama Birmingham Heersink School of Medicine clearly describes the compounds imaged in the short and long wavelength channels (77). The current FLIO version does not record the fluorescence signature of individual molecules; however, future generations of FLIO may use two-photon imaging, enabling detection of specific fluorophores involved in normal and abnormal mitochondrial, endoplasmic reticulum, and lysosomal functions (77).

**Table 1:** Retinal signal sources for fluorescence lifetime imaging ophthalmoscopy.

Fluorophore	Cell/Compartment	Captured by
Tryptophane	Membranes of photoreceptors, amacrine cells, and bipolar cells	LSC/SSC
Nicotinamide adenine dinucleotide (NADH, NADPH)	Mitochondria; in particular in the ellipsoid zone, basal RPE, and OPL	LSC/SSC
FAD	Mitochondria; in particular in the ellipsoid zone, basal RPE, and OPL	LSC/SSC
Melanin	RPE melanosomes, melanopolipofuscin, and choroidal melanocytes	LSC/SSC
Bisretinoid derivatives of vitamin A intermediates	Lysosome-related organelles (lipofuscin and melanopolipofuscin) in RPE	LSC > SSC
Collagen, elastin	Internal limiting membrane, vitreous cortex, vessels, retinal nerve fiber layer, ganglion cell layer, inner plexiform layer, inner nuclear layer, outer plexiform layer, outer nuclear layer, vessels, Bruch’s membrane	LSC/SSC
Macular xanthophyll carotenoids	Membranes of Müller glia, photoreceptors, RPE	SSC

Adapted from Goerdt et al. (77).

### Normal FLIO and proof of concept with macular telangiectasia type 2

**Figure 4:**
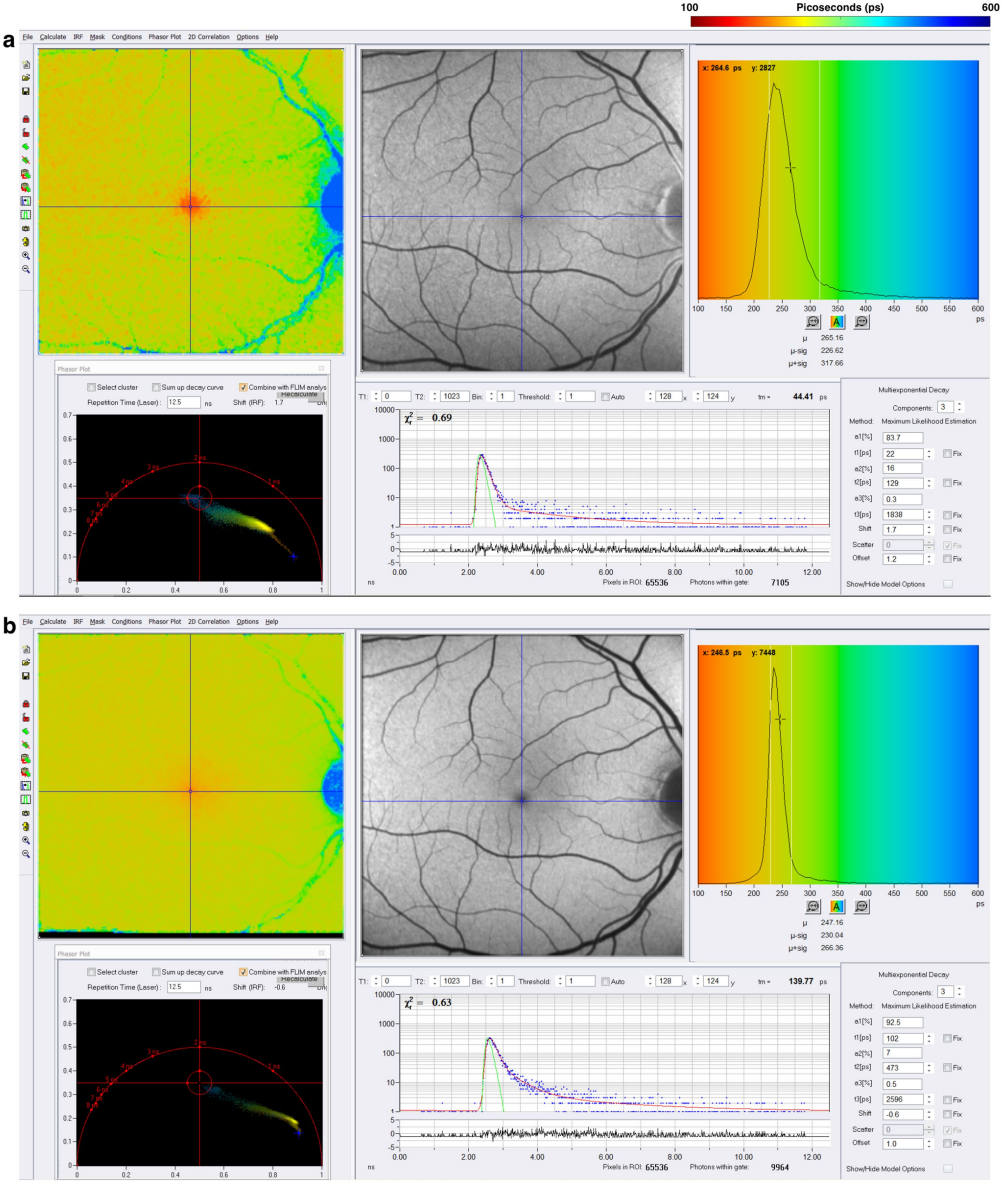
FLIO image of a healthy control; the **(a)** short spectral channel after exciting frequency of 473 nanometers [nm], capturing fluorophores with emission frequencies between 498 and 560 nm. The **(b)** long spectral channel is also excited at 473 nm but captures the emission spectrum of fluorophores within the range of 560–720 nm. Adapted from Markowitz et al. (45).

Figure 4 illustrates the normal FLIO images for the short and long spectral channels performed in a healthy control (45). FLIO data must be analyzed with control data for both gender and age, and possibly for ethnicity.

**Figure 5:**
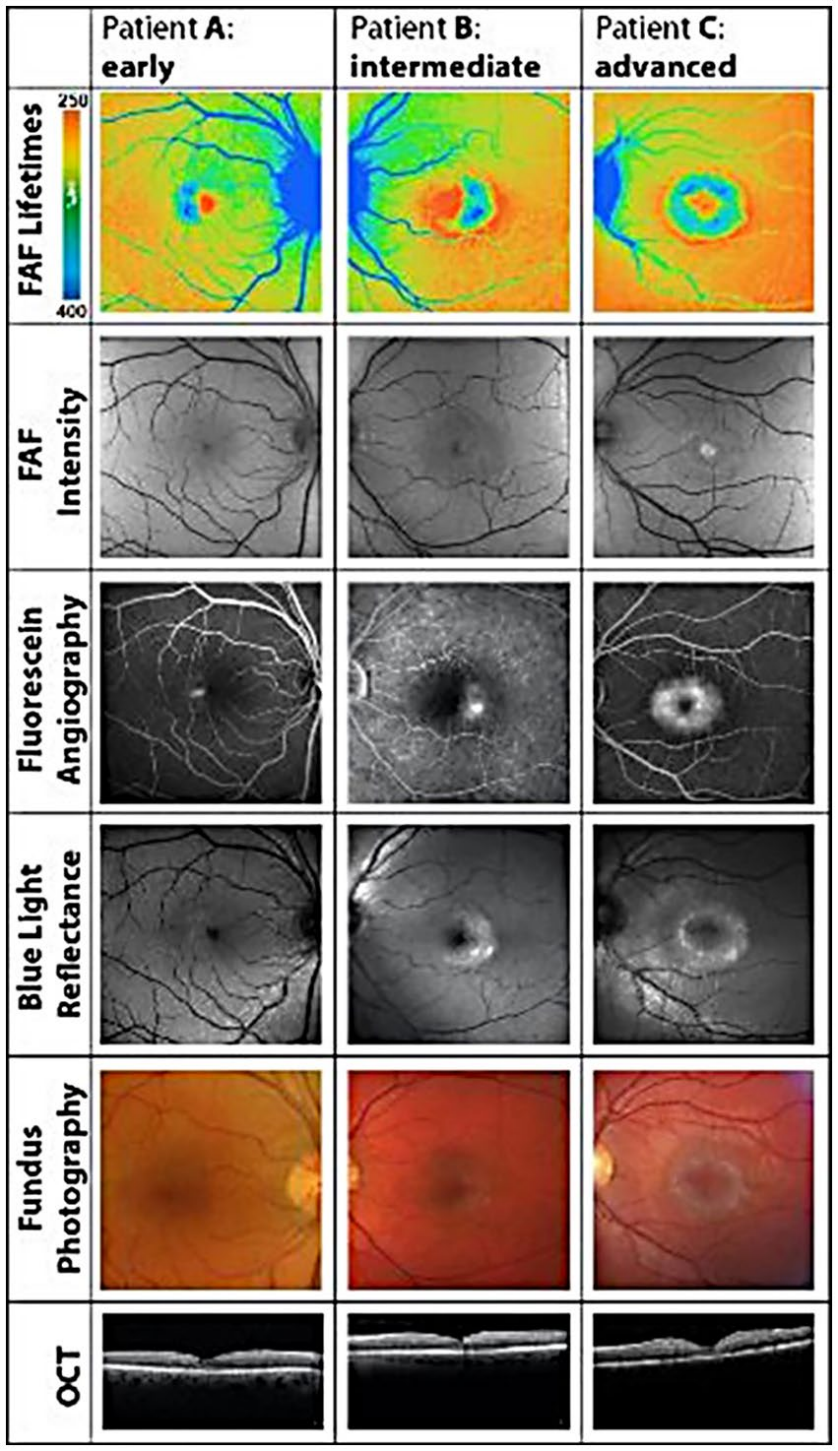
Illustrates how FLIO detects pathological changes in macular telangiectasia type 2 before any of the other retinal imaging modalities. Please compare the FLIO images in this figure to the normal FLIO images for the short spectral channel in Figure 4. Adapted from Sauer et al. (34).

Professor Paul Bernstein and his team at the University of Utah have pioneered FLIO for macular telangiectasia type 2, a rare disease, to establish this technology as an imaging biomarker whose diagnostic and longitudinal signals far exceed fundus photography, intravenous fluorescein angiography, fundus autofluorescence, and optical coherence tomography. The data from Professor Bernstein’s group is illustrated in Figure 5 (34, 37, 38).

### Analyzing FLIO data from Parkinson’s and Alzheimer’s diseases and multiple sclerosis

Because FLIO captures fluorescence lifetimes in a 256 by 256-pixel area covering a 9 by 9 mm area (35 μm x 35 μm/pixel) of the retina, targeting the macula or other areas of the retina, analyzing FLIO data is quite complex and still a work in progress. SPCImage (Becker & Hickl GmbH) is the most commonly used software for analyzing FLIO data currently. This software uses a triexponential approach to fit fluorescence decay curves at each pixel because each pixel has thousands of datum points, one data point for each time point (31–33, 39, 78, 79). The triexponential approach fits all data points into three lifetime components, each with its respective amplitude, reflecting the percent contribution of the fluorescence lifetimes in the fit model. The final output of the triexponential decay model is plotted with a color-coded heat map, representing the mean fluorescence lifetime at each pixel. This process produces colorful images of the area of the retina, where one can quickly identify areas of shorter or longer fluorescence lifetimes, providing qualitative information about the metabolic function in different regions. Each pixel from the fit model in SPCImage displays the mean fluorescence lifetime, which is useful for further quantitative analysis.

While SPCImage is the software used for calculating and fitting mean fluorescence lifetimes, post-processing analysis is helpful for investigating specific areas of each FLIO lifetime image. Additional software packages, such as FLIMX (M. Klemm, Ilmenau, Germany) and FLIO-reader (ARTORG Center for Biomedical Engineering Research, University of Bern, Switzerland), can also analyze and post-process the fluorescence lifetime data. These programs provide statistical analyses of lifetimes across and within groups of eyes. Most importantly, these programs can apply a standardized region of interest (ROI) to the FLIO images, such as the Early Treatment Diabetic Retinopathy Study (ETDRS) grid. After overlaying an ROI, such as the ETDRS grid, to each FLIO image, it is possible to quantify the means of individual lifetime components within each of the pixels that fall in a specific zone of the grid. This post-processing also allows for combining multiple areas of the ETDRS grid to identify quantitative regional fluorescence lifetime patterns from specifically defined ETDRS zones. This process, along with additional analyses, creates a more standardized and detailed approach to analyzing fluorescence lifetime data across patient eyes in various disease conditions.

### Parkinson’s disease

**Figure 6:**
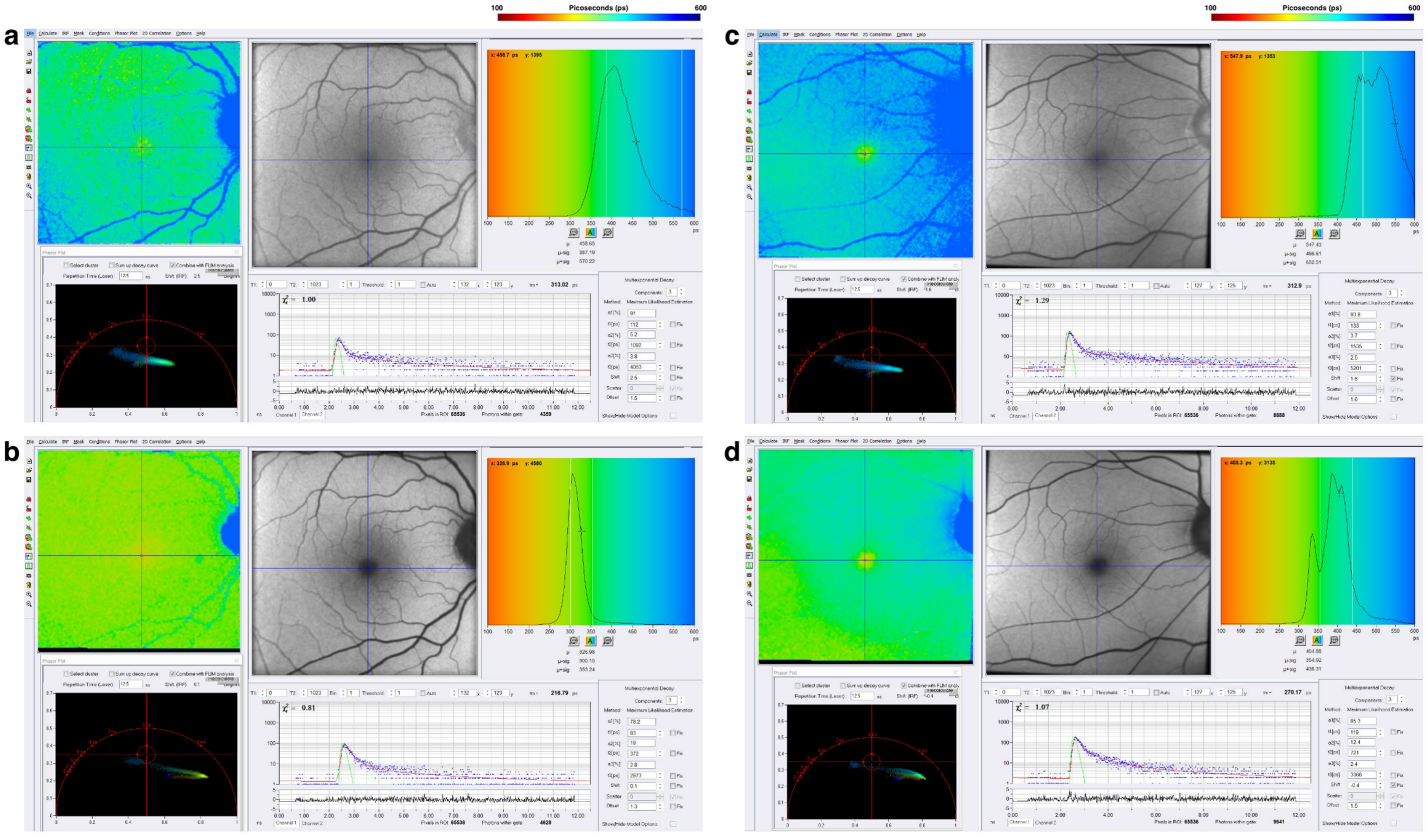
FLIO images of a patient with a 12-year diagnosis of PD showing both the **(a)** SSC and **(b)** LSC. Also shown is a patient with an 18-year diagnosis of PD showing both the **(C)** SSC and **(D)** LSC. Adapted from Markowitz et al. (45). and Shivok et al. (46).

Regardless of whether Parkinson’s develops from a variety of genetic mutations or environmental toxic exposures, such as MPTP, rotenone, and paraquat, or sporadically, the final common pathway involves dysfunction in mitochondrial ATP production, impaired mitophagy, and abnormal protein aggregation (80–84). Since one-photon FLIO captures a combination of signals that emanate in large part from FAD, NAD, and NADHP, endogenous fluorophores known to be associated with oxidative phosphorylation and mitochondrial generation of ATP, we studied a cohort of patients with Parkinson’s Disease stratified by time from the initial diagnosis. Figure 6 illustrates FLIO changes in two Parkinson’s disease patients, one 12 years after the time of diagnosis and the other 18 years after the time of diagnosis (45, 46). FLIO data in Parkinson’s Disease demonstrates prolonged fluorescence lifetimes in both the short and long spectral channels (46, 47).

### Alzheimer’s disease

**Figure 7:**
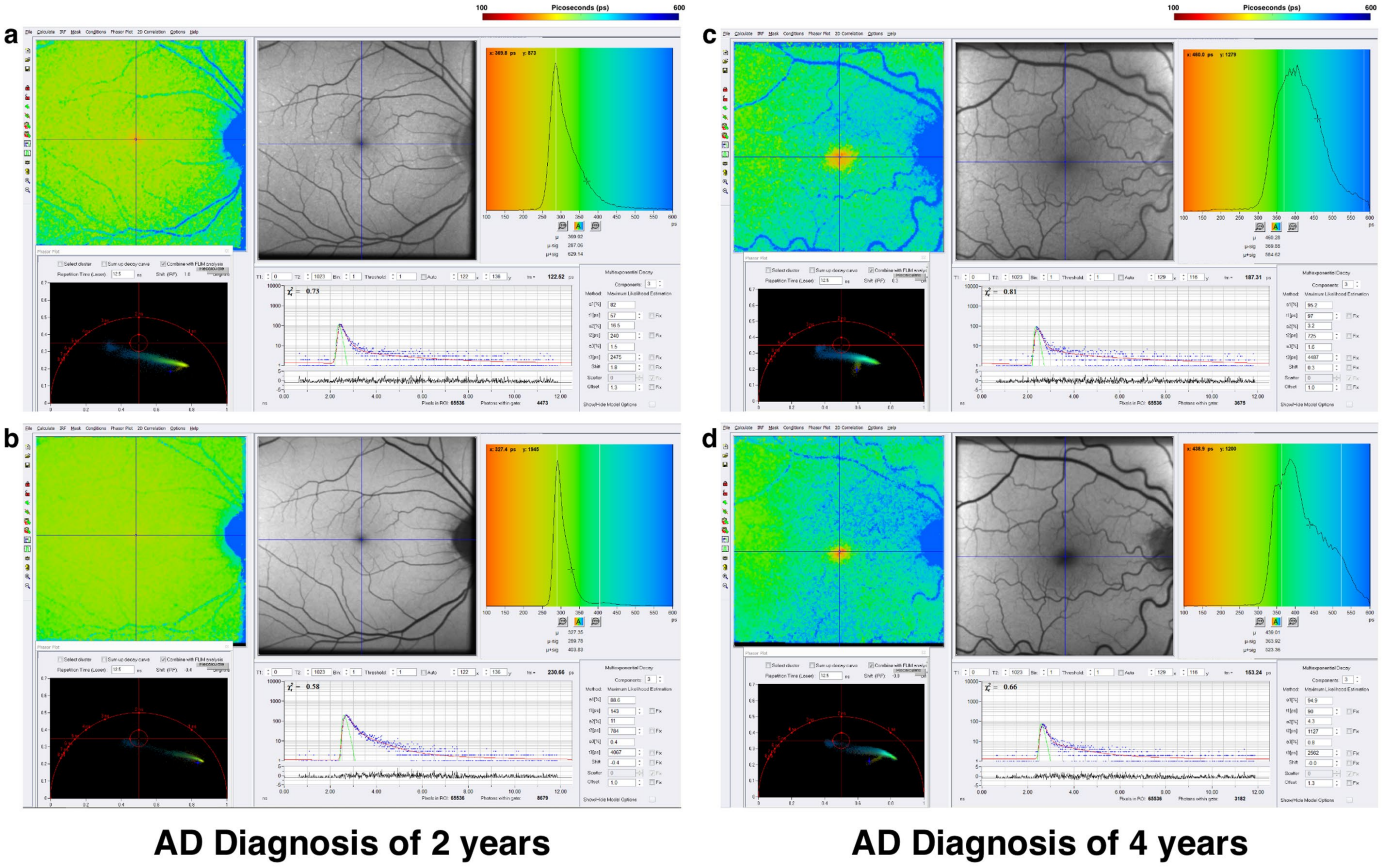
FLIO images of a patient with a 2-year diagnosis of AD showing both the **(a)** SSC and **(b)** LSC. Also shown is a patient with a 4-year diagnosis of AD showing both the **(c)** SSC and **(d)** LSC. Adapted from Markowitz et al. (45).

One-photon FLIO data in Alzheimer’s Disease demonstrates prolonged fluorescence lifetimes in both the short and long spectral channels, as seen in the comparison of Alzheimer’s disease patients with two- and four-year histories of the disease (Figure 7). Two-photon FLIO, which is now under consideration for development, may be able to determine the molecular basis for these differences in the retinal microenvironment. More and more data implicate mitochondrial dysfunction as the possible origin of Alzheimer’s Disease (85–87). Specifically, the “Mitochondrial Cascade Hypothesis” postulates that abnormal mitochondrial function impacts the processing of amyloid precursor protein (APP), a transmembrane protein, and increases Aβ oligomer production and accumulation, possibly promoting the formation of neurofibrillary tangles (88).

### Multiple sclerosis – progression independent of relapse activity “smoldering MS”

During the past 4 years, the concept and validation of progression independent of relapse activity [PIRA] or “smoldering MS” has received increasing attention in both multiple sclerosis treatment and research (89–94). PIRA asks and answers the important questions about increasing disability and transition from relapsing–remitting MS [RRMS] to secondary progressive MS [SPMS] and what serological or imaging biomarkers might predict this transition. PIRA or smoldering MS represents a distinct, disabling component of this disease, not addressed by the currently available MS medications designed to reduce clinical relapses and MRI disease activity.

Patients are classified as PIRA when they experience progressive disability without experiencing any clinical relapses, or new inflammatory lesions with MRI scanning.

**Figure 8:**
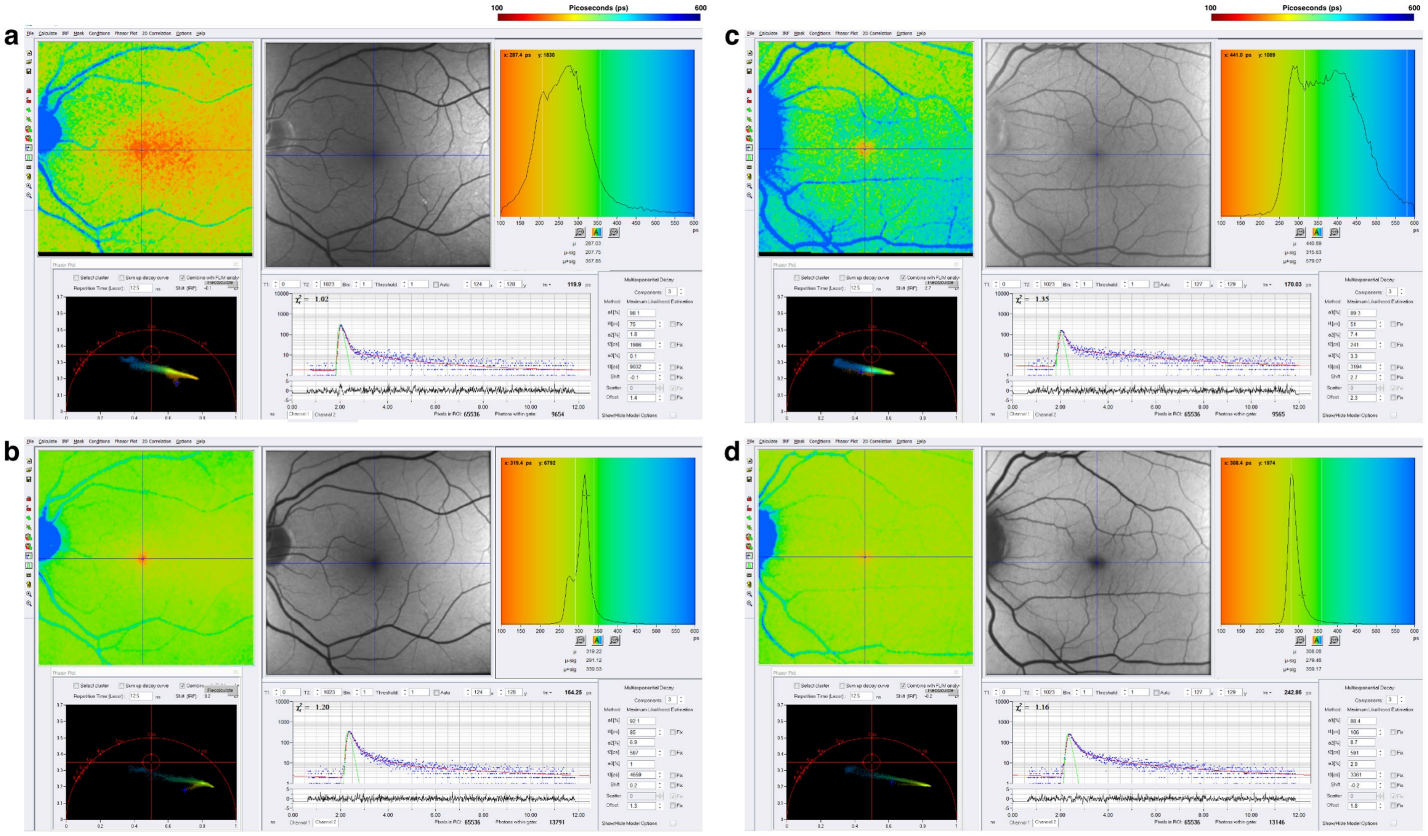
FLIO images of a patient with RRMS and 8 years of disease duration showing both the **(a)** SSC and **(b)** LSC. Also shown is a patient with SPMS and 38 years of disease duration, showing both the **(c)** SSC and **(d)** LSC. Adapted from Markowitz et al. (46).

Chronic neuroinflammation, which is below the resolution of MRI scanning, has been suggested as a pathophysiological mechanism for PIRA (90–94). Data from our group presented at the 2025 Americas Committee for Treatment and Research in Multiple Sclerosis [ACTRIMS] annual meeting suggests that mitochondrial dysfunction may also be involved (48). Figure 8 illustrates FLIO changes of a patient with RRMS and SPMS (48). FLIO data in both MS patients demonstrates prolonged fluorescence lifetimes mainly in the short spectral channel.

### Conclusion– FLIO insights and two-photon future

FLIO adds the new imaging, biomarker dimension of retinal metabolism and mitochondrial function to the existing arsenal of MRI, PET, and OCT scanning, to investigate the origins of punishing neurodegenerative syndromes, as well as a method to monitor a previously unseen disease activity.

The current one-photon FLIO setup may transition to a two-photon setup pending complete safety evaluations. Therefore, FLIO will follow the same course of MRI, CT, PET, and OCT with the images and data improving as physicists, biomedical engineers and clinical researchers unite to develop two-photon FLIO.

We anticipate that FLIO will earn an important position in the following dimensions of neurodegenerative diseases, especially when merged powerful *in vitro* methods of focused ion beam scanning electron microscopy [FIB-SEM, 3-dimensional images with 3 nanometer resolution] and fluorescence lifetime imaging microscopy [FLIM] studying retinoids and brain organoids derived from inducible pluripotential stem cells.

We foresee the following meaningful FLIO advances for patients and their families: [1] enabling an early diagnosis of disease activity especially in genetically at-risk populations or patients with early stage tremor for Parkinson’s Disease and patients with mild cognitive impairment [MCI] for Alzheimer’s Disease; [2] following the clinical course of the diseases with mitochondrial fluorophores and fluorophores of other pathologically important molecules for an early imaging biomarker of disease progression for many neurodegenerative diseases before worsening clinical symptoms and signs; and [3] discovering and evaluating in clinical trials molecules that specifically target mitochondrial dysfunction; thereby, addressing the metabolic abnormalities revealed by FLIO, as the process of “making the invisible visible” produces real-world benefits for patients, their caregivers, and their families.

### Limitations

FLIO currently faces several limitations. High cost and limited accessibility restrict its use, with fewer than 30 centers worldwide operating FLIO systems. Standardization across centers is needed, as current measurements and results cannot be directly compared between sites. Greater understanding of FLIO results, including test–retest variability, is required. The current one-photon FLIO setup does not resolve fluorescence signatures of individual molecules; however, future two-photon versions may enable detection of specific fluorophores involved in mitochondrial, endoplasmic reticulum, and lysosomal functions.

### Future directions

Future studies should be designed to observe FLIO changes longitudinally across disease stages, duration and subtypes, using larger patient cohorts and healthy controls stratified by age and sex. Developing disease-specific and healthy control reference databases, combined with AI-based analysis, will improve automated, reproducible detection of disease-related fluorescence abnormalities and enhance FLIO’s clinical utility.

## References

[1] Stoessl AJ. Neuroimaging in the early diagnosis of neurodegenerative disease. Transl Neurodegener. (2012) 1:5. doi: 10.1186/2047-9158-1-5, PMID: 23211024 PMC3506998

[2] Shimizu S Hirose D Hatanaka H Takenoshita N Kaneko Y Ogawa Y. Role of neuroimaging as a biomarker for neurodegenerative diseases. Front Neurol. (2018) 9:265. doi: 10.3389/fneur.2018.00265, PMID: 29720959 PMC5915477

[3] Haller S Jäger HR Vernooij MW Barkhof F. Neuroimaging in dementia: more than typical Alzheimer disease. Radiology. (2023) 308:e230173. doi: 10.1148/radiol.230173, PMID: 37724973

[4] Du L Roy S Wang P Li Z Qiu X Zhang Y. Unveiling the future: advancements in MRI imaging for neurodegenerative disorders. Ageing Res Rev. (2024) 95:102230. doi: 10.1016/j.arr.2024.102230, PMID: 38364912

[5] Loftus JR Puri S Meyers SP. Multimodality imaging of neurodegenerative disorders with a focus on multiparametric magnetic resonance and molecular imaging. Insights Imaging. (2023) 14:8. doi: 10.1186/s13244-022-01358-6, PMID: 36645560 PMC9842851

[6] Einarsdottir AB Hardarson SH Kristjansdottir JV Bragason DT Snaedal J Stefánsson E. Retinal oximetry imaging in Alzheimer's disease. J Alzheimers Dis. (2016) 49:79–83. doi: 10.3233/JAD-150457, PMID: 26444785

[7] Lee CS Apte RS. Retinal biomarkers of Alzheimer disease. Am J Ophthalmol. (2020) 218:337–41. doi: 10.1016/j.ajo.2020.04.040, PMID: 32387435 PMC7529847

[8] Liao C Xu J Chen Y Ip NY. Retinal dysfunction in Alzheimer’s disease and implications for biomarkers. Biomolecules. (2021) 11:1215. doi: 10.3390/biom11081215, PMID: 34439882 PMC8394950

[9] Wang X Jiao B Liu H Wang Y Hao X Zhu Y. Machine learning based on optical coherence tomography images as a diagnostic tool for Alzheimer's disease. CNS Neurosci Ther. (2022) 28:2206–17. doi: 10.1111/cns.13963, PMID: 36089740 PMC9627364

[10] Xia X Qin Q Peng Y Wang M Yin Y Tang Y. Retinal examinations provides early warning of Alzheimer's disease. J Alzheimer's Dis. (2022) 90:1341–57. doi: 10.3233/JAD-220596, PMID: 36245377

[11] Santos CY Johnson LN Sinoff SE Festa EK Heindel WC Snyder PJ. Change in retinal structural anatomy during the preclinical stage of Alzheimer’s disease. Alzheimers Dement. (2018) 10:196–209. doi: 10.1016/j.dadm.2018.01.003, PMID: 29780864 PMC5956814

[12] Chan VTT Sun Z Tang S Chen LJ Wong A Tham CC. Spectral-domain OCT measurements in Alzheimer's disease: a systematic review and Meta-analysis. Ophthalmology. (2019) 126:497–510. doi: 10.1016/j.ophtha.2018.08.009, PMID: 30114417 PMC6424641

[13] Jiang H Wang J Levin BE Baumel BS Camargo CJ Signorile JF. Retinal microvascular alterations as the biomarkers for Alzheimer disease: are we there yet? J Neuroophthalmol. (2021) 41:251–60. doi: 10.1097/WNO.0000000000001140, PMID: 33136677 PMC8079547

[14] Jiang H Wei Y Shi Y Wright CB Sun X Gregori G. Altered macular microvasculature in mild cognitive impairment and Alzheimer disease. J Neuroophthalmol. (2018) 38:292–8. doi: 10.1097/WNO.0000000000000580, PMID: 29040211 PMC5902666

[15] Biscetti L Lupidi M Luchetti E Eusebi P Gujar R Vergaro A. Novel noninvasive biomarkers of prodromal Alzheimer disease: the role of optical coherence tomography and optical coherence tomography-angiography. Eur J Neurol. (2021) 28:2185–91. doi: 10.1111/ene.14871, PMID: 33852770

[16] Grewal DS Fekrat S. Structural and functional retinal changes in preclinical Alzheimer disease. JAMA Ophthalmol. (2021) 139:556–7. doi: 10.1001/jamaophthalmol.2021.0319, PMID: 33764361

[17] Shi H Koronyo Y Rentsendorj A Fuchs D-T Sheyn J Black KL. Retinal vasculopathy in Alzheimer’s disease. Front Neurosci. (2021) 15:731614. doi: 10.3389/fnins.2021.731614, PMID: 34630020 PMC8493243

[18] Ma X Xie Z Wang H Tian Z Bi Y Li Y. A cross-sectional study of retinal vessel changes based on optical coherence tomography angiography in Alzheimer's disease and mild cognitive impairment. Front Aging Neurosci. (2023) 15:1101950. doi: 10.3389/fnagi.2023.1101950, PMID: 37113575 PMC10126258

[19] Xie J Yi Q Wu Y Zheng Y Liu Y Macerollo A. Deep segmentation of OCTA for evaluation and association of changes of retinal microvasculature with Alzheimer’s disease and mild cognitive impairment. Br J Ophthalmol. (2024) 108:432–9. doi: 10.1136/bjo-2022-321399, PMID: 36596660 PMC10894818

[20] Kaipainen A Jääskeläinen O Liu Y Haapalinna F Nykänen N Vanninen R. Cerebrospinal fluid and MRI biomarkers in neurodegenerative diseases: a retrospective memory clinic-based study. J Alzheimer's Dis. (2020) 75:751–65. doi: 10.3233/JAD-200175, PMID: 32310181 PMC7369056

[21] Coughlin DG Irwin DJ. Fluid and biopsy based biomarkers in Parkinson's disease. Neurotherapeutics. (2023) 20:932–54. doi: 10.1007/s13311-023-01379-z, PMID: 37138160 PMC10457253

[22] Delaby C Hirtz C Lehmann S. Overview of the blood biomarkers in Alzheimer's disease: promises and challenges. Rev Neurol. (2023) 179:161–72. doi: 10.1016/j.neurol.2022.09.003, PMID: 36371265

[23] Dubois B von Arnim CAF Burnie N Bozeat S Cummings J. Biomarkers in Alzheimer’s disease: role in early and differential diagnosis and recognition of atypical variants. Alzheimer's Res Ther. (2023) 15:175. doi: 10.1186/s13195-023-01314-6, PMID: 37833762 PMC10571241

[24] Faizan M Sachan N Verma O Sarkar A Rawat N Pratap SM. Cerebrospinal fluid protein biomarkers in Parkinson's disease. Clin Chim Acta. (2024) 556:117848. doi: 10.1016/j.cca.2024.117848, PMID: 38417781

[25] Ma J Tang Z Wu Y Zhang J Wu Z Huang L. Differences in blood and cerebrospinal fluid between Parkinson's disease and related diseases. Cell Mol Neurobiol. (2024) 45:9. doi: 10.1007/s10571-024-01523-z, PMID: 39729132 PMC11680620

[26] Palmqvist S Tideman P Mattsson-Carlgren N Schindler SE Smith R Ossenkoppele R. Blood biomarkers to detect Alzheimer disease in primary care and secondary care. JAMA. (2024) 332:1245–57. doi: 10.1001/jama.2024.13855, PMID: 39068545 PMC11284636

[27] Marcus C Mena E Subramaniam RM. Brain PET in the diagnosis of Alzheimer's disease. Clin Nucl Med. (2014) 39:e413–22. doi: 10.1097/RLU.0000000000000547, PMID: 25199063 PMC4332800

[28] Graff-Radford J Yong KXX Apostolova LG Bouwman FH Carrillo M Dickerson BC. New insights into atypical Alzheimer's disease in the era of biomarkers. Lancet Neurol. (2021) 20:222–34. doi: 10.1016/S1474-4422(20)30440-3, PMID: 33609479 PMC8056394

[29] Spano M Roytman M Aboian M Saboury B Franceschi A Chiang GC. Brain PET imaging: approach to cognitive impairment and dementia. PET Clin. (2023) 18:103–13. doi: 10.1016/j.cpet.2022.09.006, PMID: 36442959 PMC9713600

[30] Rabinovici GD Knopman DS Arbizu J Benzinger TLS Donohoe KJ Hansson O. Updated appropriate use criteria for amyloid and tau PET: a report from the Alzheimer's Association and Society for Nuclear Medicine and Molecular Imaging Workgroup. Alzheimers Dement. (2025) 21:e14338. doi: 10.1002/alz.14338, PMID: 39776249 PMC11772739

[31] Dysli C Quellec G Abegg M Menke MN Wolf-Schnurrbusch U Kowal J. Quantitative analysis of fluorescence lifetime measurements of the macula using the fluorescence lifetime imaging ophthalmoscope in healthy subjects. Invest Ophthalmol Vis Sci. (2014) 55:2106–13. doi: 10.1167/iovs.13-13627, PMID: 24569585

[32] Sauer L Andersen KM Dysli C Zinkernagel MS Bernstein PS Hammer M. Review of clinical approaches in fluorescence lifetime imaging ophthalmoscopy. J Biomed Opt. (2018) 23:1–20. doi: 10.1117/1.JBO.23.9.091415, PMID: 30182580 PMC8357196

[33] Sauer L Andersen KM Li B Gensure RH Hammer M Bernstein PS. Fluorescence lifetime imaging ophthalmoscopy (FLIO) of macular pigment. Invest Ophthalmol Vis Sci. (2018) 59:3094–103. doi: 10.1167/iovs.18-23886, PMID: 30025128 PMC6009392

[34] Sauer L Gensure RH Hammer M Bernstein PS. Fluorescence lifetime imaging ophthalmoscopy: a novel way to assess macular telangiectasia type 2. Ophthalmol Retina. (2018) 2:587–98. doi: 10.1016/j.oret.2017.10.008, PMID: 30116796 PMC6089530

[35] Sadda SR Borrelli E Fan W Ebraheem A Marion KM Harrington M. A pilot study of fluorescence lifetime imaging ophthalmoscopy in preclinical Alzheimer’s disease. Eye. (2019) 33:1271–9. doi: 10.1038/s41433-019-0406-2, PMID: 30923356 PMC7005755

[36] Walters S Feeks JA Huynh KT Hunter JJ. Adaptive optics two-photon excited fluorescence lifetime imaging ophthalmoscopy of photoreceptors and retinal pigment epithelium in the living non-human primate eye. Biomed Opt Express. (2022) 13:389–407. doi: 10.1364/BOE.444550, PMID: 35154879 PMC8803039

[37] Sauer L Vitale AS Andersen KM Hart B Bernstein PS. Fluorescence lifetime imaging ophthalmoscopy (FLIO) patterns in clinically unaffected children of macular telangiectasia type 2 (MACTEL) patients. Retina. (2020) 40:695–704. doi: 10.1097/IAE.0000000000002646, PMID: 31517727 PMC7062574

[38] Solberg Y Dysli C Wolf S Zinkernagel MS. Fluorescence lifetime patterns in macular telangiectasia type 2. Retina. (2020) 40:99–108. doi: 10.1097/IAE.0000000000002411, PMID: 30664123 PMC6924947

[39] Sauer L Vitale AS Modersitzki NK Bernstein PS. Fluorescence lifetime imaging ophthalmoscopy: autofluorescence imaging and beyond. Eye (Lond). (2021) 35:93–109. doi: 10.1038/s41433-020-01287-y, PMID: 33268846 PMC7852552

[40] Kunala K Tang JAH Parkins K Hunter JJ. Multispectral label-free in vivo cellular imaging of human retinal pigment epithelium using adaptive optics fluorescence lifetime ophthalmoscopy improves feasibility for low emission analysis and increases sensitivity for detecting changes with age and eccentricity. J Biomed Opt. (2024) 29:S22707. doi: 10.1117/1.JBO.29.S2.S22707, PMID: 38962492 PMC11221116

[41] Baker ML Hand PJ Wang JJ Wong TY. Retinal signs and stroke: revisiting the link between the eye and brain. Stroke. (2008) 39:1371–9. doi: 10.1161/STROKEAHA.107.496091, PMID: 18309171

[42] Hart NJ Koronyo Y Black KL Koronyo-Hamaoui M. Ocular indicators of Alzheimer's: exploring disease in the retina. Acta Neuropathol. (2016) 132:767–87. doi: 10.1007/s00401-016-1613-6, PMID: 27645291 PMC5106496

[43] Lee JY Kim JP Jang H Kim J Kang SH Kim JS. Optical coherence tomography angiography as a potential screening tool for cerebral small vessel diseases. Alzheimer's Res Ther. (2020) 12:73. doi: 10.1186/s13195-020-00638-x, PMID: 32527301 PMC7291486

[44] Olivares Ordoñez MA Smith RC Yiu G Liu YA. Retinal microstructural and microvascular changes in Alzheimer disease: a review. Int Ophthalmol Clin. (2025) 65:59–67. doi: 10.1097/IIO.0000000000000549, PMID: 39710907 PMC11817161

[45] Markowitz DM Affel E Hajnóczky G Sergott RC. Future applications of fluorescence lifetime imaging ophthalmoscopy in neuro-ophthalmology, neurology, and neurodegenerative conditions. Front Neurol. (2025) 16:1493876. doi: 10.3389/fneur.2025.1493876, PMID: 40125394 PMC11927091

[46] Shivok K Affel E Alizadeh M Liang T Kremens D Sergott RC. International congress of Parkinson’s disease and movement disorders; august 28, 2023. Copenhagen: Denmark (2023).

[47] Shivok K Affel E Alizadeh M Liang T-W Kremens K Sergott R. Fluorescence lifetime imaging ophthalmoscopy (FLIO), a novel retinal mitochondrial biomarker for Parkinson’s disease. Mov Disord. (2024) 39:1. doi: 10.1002/alz.0918803829404638294046

[48] Markowitz DM Shivok K Affel E Pulido JS Leist T Hajnoczky G. Fluorescence lifetime imaging ophthalmoscopy (FLIO): A novel retinal metabolic biomarker for multiple sclerosis. Florida: West Palm Beach (2025).

[49] Crutch SJ Isaacs R Rossor MN. Some workmen can blame their tools: artistic change in an individual with Alzheimer's disease. Lancet. (2001) 357:2129–33. doi: 10.1016/S0140-6736(00)05187-4, PMID: 11445128

[50] About William Utermohlen. *About William Utermohlen*. Available online at: https://www.williamutermohlen.com/about.

[51] Moons L De Groef L. Multimodal retinal imaging to detect and understand Alzheimer’s and Parkinson’s disease. Curr Opin Neurobiol. (2022) 72:1–7. doi: 10.1016/j.conb.2021.07.007, PMID: 34399146

[52] Cao Q Yang S Wang X Sun H Chen W Wang Y. Transport of β-amyloid from brain to eye causes retinal degeneration in Alzheimer's disease. J Exp Med. (2024) 221:386. doi: 10.1084/jem.20240386, PMID: 39316084 PMC11448872

[53] Gaire BP Koronyo Y Fuchs D-T Shi H Rentsendorj A Danziger R. Alzheimer's disease pathophysiology in the retina. Prog Retin Eye Res. (2024) 101:101273. doi: 10.1016/j.preteyeres.2024.101273, PMID: 38759947 PMC11285518

[54] Den Haan J Morrema THJ Verbraak FD de Boer JF Scheltens P Rozemuller AJ. Amyloid-beta and phosphorylated tau in post-mortem Alzheimer’s disease retinas. Acta Neuropathol Commun. (2018) 6:147. doi: 10.1186/s40478-018-0650-x, PMID: 30593285 PMC6309096

[55] Cheung CY Chan VTT Mok VC Chen C Wong TY. Potential retinal biomarkers for dementia: what is new? Curr Opin Neurol. (2019) 32:82–91. doi: 10.1097/WCO.0000000000000645, PMID: 30566412

[56] Cheung CY Ikram MK Chen C Wong TY. Imaging retina to study dementia and stroke. Prog Retin Eye Res. (2017) 57:89–107. doi: 10.1016/j.preteyeres.2017.01.001, PMID: 28057562

[57] Lee MJ Abraham AG Swenor BK Sharrett AR Ramulu PY. Application of optical coherence tomography in the detection and classification of cognitive decline. J Curr Glaucoma Pract. (2018) 12:10–8. doi: 10.5005/jp-journals-10028-1238, PMID: 29861577 PMC5981088

[58] Hansen DV Hanson JE Sheng M. Microglia in Alzheimer's disease. J Cell Biol. (2018) 217:459–72. doi: 10.1083/jcb.201709069, PMID: 29196460 PMC5800817

[59] Xu Y Gao W Sun Y Wu M. New insight on microglia activation in neurodegenerative diseases and therapeutics. Front Neurosci. (2023) 17:1308345. doi: 10.3389/fnins.2023.1308345, PMID: 38188026 PMC10770846

[60] Cheung CY Ong YT Ikram MK Ong SY Li X Hilal S. Microvascular network alterations in the retina of patients with Alzheimer's disease. Alzheimers Dement. (2014) 10:135–42. doi: 10.1016/j.jalz.2013.06.009, PMID: 24439169

[61] O'Bryhim BE Apte RS Kung N Coble D Van Stavern GP. Association of Preclinical Alzheimer Disease with Optical Coherence Tomographic Angiography Findings. JAMA Ophthalmol. (2018) 136:1242–8. doi: 10.1001/jamaophthalmol.2018.3556, PMID: 30352114 PMC6248182

[62] La Morgia C Ross-Cisneros FN Koronyo Y Hannibal J Gallassi R Cantalupo G. Melanopsin retinal ganglion cell loss in Alzheimer disease. Ann Neurol. (2016) 79:90–109. doi: 10.1002/ana.24548, PMID: 26505992 PMC4737313

[63] La Morgia C Ross-Cisneros FN Sadun AA Carelli V. Retinal ganglion cells and circadian rhythms in Alzheimer’s disease, Parkinson's disease, and beyond. Front Neurol. (2017) 8:162. doi: 10.3389/fneur.2017.00162, PMID: 28522986 PMC5415575

[64] Steiner OL de Zeeuw J. Melanopsin retinal ganglion cell function in Alzheimer's vs. Parkinson's disease an exploratory meta-analysis and review of pupillometry protocols. Parkinsonism Relat Disord. (2024) 123:106063. doi: 10.1016/j.parkreldis.2024.106063, PMID: 38443213

[65] Doustar J Torbati T Black KL Koronyo Y Koronyo-Hamaoui M. Optical coherence tomography in Alzheimer’s disease and other neurodegenerative diseases. Front Neurol. (2017) 8:701. doi: 10.3389/fneur.2017.00701, PMID: 29312125 PMC5742098

[66] Gupta VB Chitranshi N den Haan J Mirzaei M You Y Lim JK. Retinal changes in Alzheimer's disease- integrated prospects of imaging, functional and molecular advances. Prog Retin Eye Res. (2021) 82:100899. doi: 10.1016/j.preteyeres.2020.100899, PMID: 32890742

[67] Ashraf G McGuinness M Khan MA Obtinalla C Hadoux X van Wijngaarden P. Retinal imaging biomarkers of Alzheimer's disease: a systematic review and meta-analysis of studies using brain amyloid beta status for case definition. Alzheimers Dement (Amst). (2023) 15:e12421. doi: 10.1002/dad2.12421, PMID: 37250908 PMC10210353

[68] Granger CE Yang Q Song H Saito K Nozato K Latchney LR. Human retinal pigment epithelium: in vivo cell morphometry, multispectral autofluorescence, and relationship to cone mosaic. Invest Ophthalmol Vis Sci. (2018) 59:5705–16. doi: 10.1167/iovs.18-24677, PMID: 30513531 PMC6280915

[69] Papour A Taylor Z Stafsudd O Tsui I Grundfest W. Imaging autofluorescence temporal signatures of the human ocular fundus in vivo. J Biomed Opt. (2015) 20:110505. doi: 10.1117/1.JBO.20.11.110505, PMID: 26590217

[70] Chen AX Conti TF Hom GL Greenlee TE Raimondi R Briskin IN. Functional imaging of mitochondria in retinal diseases using flavoprotein fluorescence. Eye (Lond). (2021) 35:74–92. doi: 10.1038/s41433-020-1110-y, PMID: 32709959 PMC7852520

[71] Caro R Chen A Mudumbai R Duerr E Chen PP Bojikian KD. In vivo imaging of mitochondrial function in normal, glaucoma suspect, and glaucoma eyes. PLoS One. (2025) 20:e0317354. doi: 10.1371/journal.pone.0317354, PMID: 39808681 PMC11731742

[72] Boguslawski J Palczewska G Tomczewski S Milkiewicz J Kasprzycki P Stachowiak D. In vivo imaging of the human eye using a 2-photon-excited fluorescence scanning laser ophthalmoscope. J Clin Invest. (2022) 132:218. doi: 10.1172/JCI154218, PMID: 34847075 PMC8759795

[73] Tang JAH Granger CE Kunala K Parkins K Huynh KT Bowles-Johnson K. Adaptive optics fluorescence lifetime imaging ophthalmoscopy of in vivo human retinal pigment epithelium. Biomed Opt Express. (2022) 13:1737–54. doi: 10.1364/BOE.451628, PMID: 35414970 PMC8973160

[74] Gao X Yang L Petros JA Marshall FF Simons JW Nie S. In vivo molecular and cellular imaging with quantum dots. Curr Opin Biotechnol. (2005) 16:63–72. doi: 10.1016/j.copbio.2004.11.003, PMID: 15722017

[75] Fleming KG. Fluorescence theory In: Lindon JC, editor. Encyclopedia of spectroscopy and spectrometry. 2nd ed. Oxford: Academic Press (2010). 628–34.

[76] Ghasemi F Fahimi-Kashani N Bigdeli A Alshatteri AH Abbasi-Moayed S Al-Jaf SH. Paper-based optical nanosensors – a review. Anal Chim Acta. (2023) 1238:340640. doi: 10.1016/j.aca.2022.340640, PMID: 36464453

[77] Goerdt L Clark ME Thomas TN Gao L McGwin G Jr Hammer M. Fluorescence lifetime imaging ophthalmoscopy, vision, and Chorioretinal asymmetries in aging and age-related macular degeneration: ALSTAR2. Invest Ophthalmol Vis Sci. (2025) 66:56. doi: 10.1167/iovs.66.4.56, PMID: 40257785 PMC12020951

[78] Schweitzer D Schenke S Hammer M Schweitzer F Jentsch S Birckner E. Towards metabolic mapping of the human retina. Microsc Res Tech. (2007) 70:410–9. doi: 10.1002/jemt.20427, PMID: 17393496

[79] Klemm M Schweitzer D Peters S Sauer L Hammer M Haueisen J. FLIMX: a software package to determine and analyze the fluorescence lifetime in time-resolved fluorescence data from the human eye. PLoS One. (2015) 10:e0131640. doi: 10.1371/journal.pone.0131640, PMID: 26192624 PMC4507995

[80] Tysnes OB Storstein A. Epidemiology of Parkinson's disease. J Neural Transm (Vienna). (2017) 124:901–5. doi: 10.1007/s00702-017-1686-y, PMID: 28150045

[81] Cabreira V Massano J. Parkinson's disease: clinical review and update. Acta Medica Port. (2019) 32:661–70. doi: 10.20344/amp.11978, PMID: 31625879

[82] Cerri S Mus L Blandini F. Parkinson's disease in women and men: what's the difference? J Parkinsons Dis. (2019) 9:501–15. doi: 10.3233/JPD-191683, PMID: 31282427 PMC6700650

[83] Reich SG Savitt JM. Parkinson's disease. Med Clin North Am. (2019) 103:337–50. doi: 10.1016/j.mcna.2018.10.014, PMID: 30704685

[84] Morris HR Spillantini MG Sue CM Williams-Gray CH. The pathogenesis of Parkinson's disease. Lancet. (2024) 403:293–304. doi: 10.1016/S0140-6736(23)01478-2, PMID: 38245249

[85] Wang W Zhao F Ma X Perry G Zhu X. Mitochondria dysfunction in the pathogenesis of Alzheimer’s disease: recent advances. Mol Neurodegener. (2020) 15:30. doi: 10.1186/s13024-020-00376-6, PMID: 32471464 PMC7257174

[86] Misrani A Tabassum S Yang L. Mitochondrial dysfunction and oxidative stress in Alzheimer’s disease. Frontiers in aging. Neuroscience. (2021) 13:617588. doi: 10.3389/fnagi.2021.617588, PMID: 33679375 PMC7930231

[87] Wang S Liao Z Zhang Q Han X Liu C Wang J. Mitochondrial dysfunction in Alzheimer's disease: a key frontier for future targeted therapies. Front Immunol. (2024) 15:1484373. doi: 10.3389/fimmu.2024.1484373, PMID: 39877373 PMC11772192

[88] Swerdlow RH Burns JM Khan SM. The Alzheimer's disease mitochondrial cascade hypothesis. J Alzheimers Dis. (2010) 20:S265–79. doi: 10.3233/JAD-2010-100339, PMID: 20442494 PMC2883665

[89] Lublin FD Häring DA Ganjgahi H Ocampo A Hatami F Čuklina J. How patients with multiple sclerosis acquire disability. Brain. (2022) 145:3147–61. doi: 10.1093/brain/awac016, PMID: 35104840 PMC9536294

[90] Müller J Cagol A Lorscheider J Tsagkas C Benkert P Yaldizli Ö. Harmonizing definitions for progression independent of relapse activity in multiple sclerosis: a systematic review. JAMA Neurol. (2023) 80:1232–45. doi: 10.1001/jamaneurol.2023.3331, PMID: 37782515

[91] Tur C Carbonell-Mirabent P Cobo-Calvo Á Otero-Romero S Arrambide G Midaglia L. Association of Early Progression Independent of relapse activity with long-term disability after a first demyelinating event in multiple sclerosis. JAMA Neurol. (2023) 80:151–60. doi: 10.1001/jamaneurol.2022.4655, PMID: 36534392 PMC9856884

[92] Calabrese M Preziosa P Scalfari A Colato E Marastoni D Absinta M. Determinants and biomarkers of progression independent of relapses in multiple sclerosis. Ann Neurol. (2024) 96:1–20. doi: 10.1002/ana.26913, PMID: 38568026

[93] Ciccarelli O Barkhof F Calabrese M De Stefano N Eshaghi A Filippi M. Using the progression independent of relapse activity framework to unveil the pathobiological foundations of multiple sclerosis. Neurology. (2024) 103:e209444. doi: 10.1212/WNL.0000000000209444, PMID: 38889384 PMC11226318

[94] Tur C Rocca MA. Progression independent of relapse activity in multiple sclerosis: closer to solving the pathologic puzzle. Neurology. (2024) 102:e207936. doi: 10.1212/WNL.0000000000207936, PMID: 38165383

